# Residual Strength Prediction of Aluminum Panels with Multiple Site Damage Using Artificial Neural Networks

**DOI:** 10.3390/ma13225216

**Published:** 2020-11-18

**Authors:** Ala Hijazi, Sameer Al-Dahidi, Safwan Altarazi

**Affiliations:** 1 Department of Mechanical Engineering, German Jordanian University, Amman 11180, Jordan; sameer.aldahidi@gju.edu.jo; 2 Department of Industrial Engineering, German Jordanian University, Amman 11180, Jordan; safwan.altarazi@gju.edu.jo

**Keywords:** fracture, multiple site damage cracks, residual strength, aircraft fuselage panels, stiffened panels, lap-joint panels, artificial neural networks, ANN optimization

## Abstract

Multiple site damage (MSD) cracks are small fatigue cracks that may accumulate at the sides of highly loaded holes in aging aircraft structures. The presence of MSD cracks can drastically reduce the residual strength of fuselage panels. In this paper, artificial neural networks (ANN) modeling is used for predicting the residual strength of aluminum panels with MSD cracks. Experimental data that include 147 unique configurations of aluminum panels with MSD cracks are used. The experimental dataset includes three different aluminum alloys (2024-T3, 2524-T3, and 7075-T6), four different test panel configurations (unstiffened, stiffened, stiffened with a broken middle stiffener, and bolted lap-joints), many different panel widths and thicknesses, and the sizes of the lead and MSD cracks. The results presented in this paper demonstrate that a single ANN model can predict the residual strength for all materials and configurations with high accuracy. Specifically, the overall mean absolute error for the ANN model predictions is 3.82%. Furthermore, the ANN model residual strength predictions are compared to those obtained using the most accurate semi-analytical and computational approaches from the literature. The ANN model predictions are found to be at the same accuracy level of these approaches, and they even outperform the other approaches for many configurations.

## 1. Introduction

The concern about multiple site damage (MSD) typically arises for aging passenger and transport aircraft, especially since many of these aircraft are being used beyond their original design life. MSD cracks are small fatigue cracks that may accumulate at the sides of highly loaded holes (rivet holes in particular) in the aircraft’s fuselage or internal structure. These MSD cracks usually appear after an extended period of time due to the large number of loading cycles. Aircraft manufacturers design the fuselage of their airplanes to be able to carry the design load with the presence of a relatively large crack (in the fuselage) spanning several adjacent rivet holes. However, the presence of MSD cracks can significantly reduce the structure’s ability to carry loads [1,2,3]. The significant effect of the MSD phenomenon on aircraft’s structural integrity was brought to light after the Aloha Airlines (flight 243) incident in 1988 where a large section of the upper crown structure was separated from the fuselage in midair. From that time onwards, the MSD phenomenon started to gain attention. Nowadays, the inspection for MSD cracks is part of the aircraft’s maintenance procedures, and aircraft manufacturers take MSD into consideration when designing their airplanes. In general, there are three concerns related to the MSD phenomenon: crack initiation, crack growth life, and residual strength of panels with MSD cracks. The residual strength of a cracked structure simply refers to the maximum stress level (or load) the structure can withstand before fracture. In the case that MSD cracks are present along with a lead crack, residual strength is usually used to refer to the stress level at which the ligaments between the lead crack and adjacent MSD cracks on both sides collapse. The collapse of these ligaments, which is usually referred to as linkup, will cause the entire panel to fracture, unless crack arresting structures (such as stiffeners) are used. Therefore, the residual strength of panels with MSD is also sometimes referred to as linkup stress.

Many researchers have investigated the ability of several methodologies to estimate the residual strength of panels with MSD, and the accuracy of these methodologies varied substantially. The methodologies ranged all the way from simple engineering models to sophisticated, robust computational techniques. The vast majority of these methodologies relied on analytical or semi-analytical (empirically corrected) models [1,2,3,4,5,6,7,8,9,10,11,12,13,14,15,16,17]. Nevertheless, other methodologies such as elastic–plastic finite element analysis (FEA) along with the crack tip opening angle (CTOA) criterion [12,18], weight functions combined with CTOA [19], system reliability method [20], and computational intelligence techniques [21,22] were also used for residual strength estimation for panels with MSD.

The artificial neural networks (ANN) is one of the advanced computational intelligence methods, which is inspired by the way the human nervous system works. The ANN resembles the work principle of the human brain by acquiring knowledge through a learning process and storing this knowledge through interneuron connections of different synaptic weight [23]. Based on past experience gained from the input/output dataset through the training process, an ANN learns how the system behaves, and based on that, it can predict the outputs for any new set of inputs. The ANN’s ability to learn by examples make them particularly useful for modeling highly complicated and nonlinear processes where the development of thorough analytical models is extremely difficult. The ANNs are usually built directly from experimental data without the need for any prior knowledge about the relations between the input/output parameters. The use of ANNs has always been seen as a simple and attractive alternative approach to using some of the complicated analytical or computational models. In general, the greatest advantage of ANNs is its ability to model complex nonlinear relationships between several input/output parameters without any prior knowledge of the nature of the relationships between these parameters.

Since its introduction in the 1960s, ANNs continued to provide a powerful framework for modeling nonlinear systems, and they were used in a wide variety of engineering applications, including automatic control [24], solar energy systems [25], traffic and transportation [26], image processing [27], optimization of structures [28], materials science and engineering [29,30,31,32], manufacturing [33], fracture mechanics, and fault detection [34,35,36,37,38,39,40,41,42,43,44,45,46,47,48,49,50,51]. In fracture mechanics, ANNs were mostly used in applications concerned with crack propagation, fatigue life, and failure mode prediction [34]. As a matter of fact, ANNs did not find much use in the field of mechanical fracture and fracture parameters as in other fields. This can mainly be attributed to the nature of this field, where it is not easy to generate large experimental datasets for training the ANN due to practical constraints related to the time and cost requirements in many mechanical fracture experiments. Some researchers have employed ANN for predicting some fracture parameters for different materials. Seibi and Al-Alawi [46] employed ANN to predict the fracture toughness in beams and plates made of aluminum under uniaxial and biaxial loading. In addition, Ince [47], used ANN to predict the stress intensity factor and crack tip opening displacement for concrete. The use of ANN for residual strength predictions of aircraft panels with MSD is actually very rare in the literature. This is most likely due to the somewhat limited number of available experimental data for this type of problem along with the variety of possible test panel configurations (unstiffened, stiffened, lap-joint, curved, etc.) and materials. Pidaparti et al. [21,22] used ANN for predicting the corrosion rate and residual strength of unstiffened aluminum panels with corrosion thinning and MSD. About 40 experimental data points, obtained from the literature, were used for training the network, and the network was tested using a selected group of nine unstiffened panels. The ANN residual strength predictions were compared to the experimental results, and the mean absolute error was found to be about 12%. In fact, such an error level is considered to be relatively high compared to some of the other simple engineering models reported in the literature [9].

Previous experimental investigations done by Smith et al. [9,13,14] led to the development of relatively simple to use semi-analytical models (i.e., empirically corrected analytical models) for the three aluminum alloys commonly used in the aircraft industry (2024-T3, 2524-T3, and 7075-T6). These models give fairly accurate residual strength predictions. However, they require using some geometric correction factors (usually obtained from charts), and sometimes, it will be necessary to use FEA to obtain some of these geometric correction factors. Therefore, it is desirable to develop an ANN model for obtaining quick and accurate residual strength predictions. It will also be advantageous if a single ANN model can be used for the different panel materials and geometric configurations. In this paper, we use ANN modeling for estimating the residual strength of aluminum panels with MSD. Our results demonstrate that a single ANN model can predict the residual strength for the different panel materials and configurations that are being considered here. The experimental MSD residual strength data used in this investigation are obtained from several sources in the literature [7,8,9,10,11,13,14,19]. A total of 147 unique data points are used here where the data includes three different aluminum sheet materials (2024-T3, 2524-T3, 7075-T6), four different panel configurations (unstiffened, stiffened, stiffened with middle broken stiffener, bolted lap-joint), different panel widths, sheet thickness, and grain orientation, along with many different lead and MSD cracks geometries. The 147 data points are split into three groups: training, validation, and testing, where the data points in each group are chosen randomly. In order to get more reliable results, the random selection of the data points in the training, validation, and testing datasets is repeated 40 times, and ANN models are developed using each of these 40 random combinations of training, validation, and testing datasets. A total of 97 data points that covered all the different configurations (materials, panel configuration, etc.) are used for training the ANN, while 50 data points are retained for validation and testing (25 each). The training datasets are used to develop/build the ANN prediction model, while the validation datasets are used to optimize the ANN’s configuration in terms of the number of hidden nodes (i.e., neurons), back-propagation learning algorithm, and hidden nodes activation function; and finally, the testing datasets (unseen previously by the ANN) are used to evaluate the performance of the built-ANN predictions with respect to the actual experimental values. A feed-forward ANN model having one hidden layer with nine independent inputs and one output (residual strength) is used in this investigation. The nine inputs used for the ANN model cover the panel and cracks geometry, the panel material and panel configuration. The ANN is optimized to give the best performance based on two performance metrics: the mean absolute percentage error (MAE_P_) and the root mean square percentage error (RMSE_P_). The ANN optimization covered three of the most commonly used learning algorithms, 12 different activation functions and up to 30 nodes in the hidden layer. Based on this optimization, the Bayesian Regularization (BR) learning algorithm, the Elliot symmetric sigmoid activation function, and seven hidden nodes are used in the ANN model. Our results show that the ANN is able to predict the residual strength for all the different materials and panel configurations with a mean absolute error of about 3.82%. The results also show that the ANN predictions are generally accurate for all the different materials and panel configurations. The obtained ANN predictions are also compared with the residual strength predictions of the best available fracture mechanics semi-analytical and computational models. The comparison shows that the ANN results are of comparable accuracy and even give more accurate results in many cases.

## 2. Background 

### 2.1. Residual Strength of Panels with MSD

**Figure 1:**
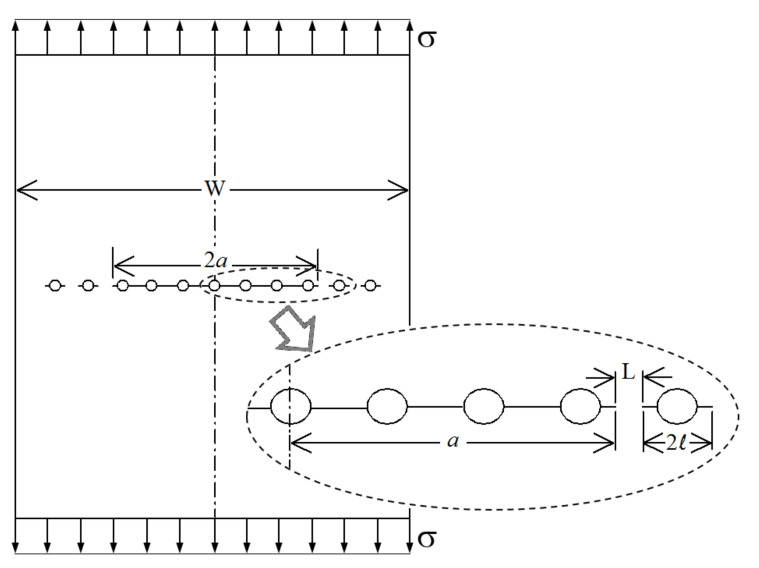
Illustration of the multiple site damage (MSD) test panel geometry definitions.

The residual strength of panels having a lead crack along with adjacent MSD cracks, as illustrated in Figure 1, can theoretically be predicted using different analytical theories. The Linear Elastic Fracture Mechanics (LEFM) is one of the most fundamental theories that can be used in a variety of fracture mechanics problems [52]. The LEFM is based on the assumption that material at the crack tip behaves linearly elastic; thus, LEFM is more applicable to brittle materials. According to LEFM, failure (or unstable crack extension) will occur when the value of the crack-tip Stress Intensity Factor (SIF) reaches a critical value. This limiting value of the SIF is called the fracture toughness (K_C_). The fracture toughness is a material property, but for thin sheets, it is also slightly dependent on the thickness, grain orientation, and crack length. Another theory that can be used for predicting the residual strength is the Net Section Yielding (NSY), and it is more applicable to ductile materials. However, experiments have shown that neither of the LEFM nor NSY theories is able to accurately predict the residual strength of panels with MSD for neither ductile nor brittle materials [13].

An analytical model that is especially formulated for the prediction of residual strength of panels with MSD was introduced by Swift [1]. This model is called the “Linkup”, model and it is based on the concept that the ligament between the lead crack and the adjacent MSD crack will fail when the remote stress reaches a level that causes the lead crack-tip plastic zone and the adjacent MSD crack-tip plastic zone to touch each other (i.e., merge together). According to the linkup model, the remote stress that causes failure of the ligament, which is called “linkup” stress (σ_LU_), is found as:

(1) $$\sigma_{\mathrm{LU}}=\sigma_{y}\sqrt{\frac{2L}{a\;\beta_{a}{}^{2}+\ell \;\beta_{\ell }{}^{2}}}$$

where

σ_y_: The yield strength of the material.
L: Length of the ligament between the lead crack and MSD crack.
*a*: Lead crack half-length.
ℓ: MSD crack half-length.
$\beta_{a}$: The overall SIF correction factor for the lead crack tip.
$\beta_{\ell }$: The overal SIF correction factor for the adjacent MSD crack tip.

The SIF geometric correction factors (usually referred to as “betas”) are usually readily available in the literature in the form of equations or charts, and in case they are not available for some configurations, they can be determined using FEA [15].

As mentioned previously, as the linkup occurs, the entire panel will fail (assuming MSD cracks exist on all subsequent holes); therefore, the linkup stress (Equation (1)) is equal to the residual strength of the panel. Experimental investigations have shown that the linkup model is not accurate for many crack configurations [2,5,7,8,9,10,11,12,13,14,15,19]. Another analytical model that shares the same basic concept with the linkup model, but is based on a different plastic zone size model (strip-yield), was introduced by Kuang and Chen [6]. This model is based on the use of an iterative approach that is not so easy to implement for engineering use, and it gave an average error of about 10% compared to their test results. In order to improve the accuracy of the residual strength predictions of Swift’s linkup model, some researchers developed empirical corrections for the linkup model based on experimental data [2,5,9,13,14]. The most accurate of these empirically corrected models is that developed by Smith et al. [9] for 2024-T3 aluminum sheets, which is referred to as the “WSU2” model. This semi-analytical model was developed based on test data of 40 unstiffened panels, and it is based on the use of the standardized A-Basis or B-Basis yield strength values (obtained from the MIL-HDBK-5H [53]). The accuracy of the “WSU2” model was further verified using test data for stiffened panels and bolted lap-joint panels [10,11]. Overall, the model was able to predict the residual strength for over 100 panels that included unstiffened, stiffened, and lap-joint panels with a mean absolute error of about 4.5% [15]. Using the same approach, two other semi-analytical models were also developed for 7075-T6 aluminum (the “MLU7075” model) and 2524-T3 aluminum (the “MLU2524” model) [13,14]. For A-Basis yield strength values and using (SI) units, the three models for 2024-T3, 2524-T3, and 7075-T6, respectively, are given as:

(2) $$\sigma_{\mathrm{WSU}2}=\frac{\sigma_{\mathrm{LU}}}{1.3123+0.3065\ln\left(L/25.4\right)}$$

(3) $$\sigma_{\mathrm{MLU}2524}=\frac{\sigma_{\mathrm{LU}}}{0.9683+0.1905\ln\left(L/25.4\right)}$$

(4) $$\sigma_{\mathrm{MLU}7075}=\frac{\sigma_{\mathrm{LU}}}{1.377+1.042\;\left(L/25.4\right)}$$

where $\sigma_{\mathrm{LU}}$ is the linkup stress (given by Equation (1)) and L is the ligament length in “millimeters”. The experimental results show that all of these three empirically corrected models can predict the residual strength with very good accuracy (3% and 2% mean absolute errors for the LU2524 and LU7075 models, respectively). Since these models are fairly accurate and, at the same time, are relatively easy to calculate, they are used in this paper to compare their residual strength predictions with the ANN predictions in terms of accuracy. 

The CTOA criterion is one of the more recent sophisticated Elastic–Plastic Fracture Mechanics (EPFM)-based techniques that can be used for predicting residual strength [52]. The assumption of this criterion is that crack growth will occur when the crack-tip opening angle reaches a critical value. A variety of numerical techniques exist to facilitate the application of the CTOA criterion. Predicting the residual strength of cracked panels based on the CTOA criterion generally requires the use of a three-dimensional elastic–plastic FEA because of the three-dimensional state of stress at the crack tip. However, for thin sheets, two-dimensional FEA can be used if a thin layer of plane stain elements is placed along the crack plane to account for the higher constraint at the crack tip [54]. Dawicke and Newman [18] used three-dimensional FEA along with the CTOA criterion for predicting the stable crack extension and residual strength of panels with MSD and reported that the simulation results were within 7% of the experimental measurements. Hijazi et al. [12] used two-dimensional FEA along with the CTOA criterion for predicting the residual strength of bolted lap-joint panels with MSD and reported that the simulations were able to predict the residual strength with about 3% mean absolute error. Although the elastic–plastic FEA analysis along with the CTOA criterion can generally predict residual strength with very reasonable accuracy, such approach is time consuming and not very suitable for engineering use. Xu et al. [19] proposed a unifying method for solving problems involving collinear cracks. Their method combines the CTOA criterion with crack weight function and strip-yield plastic zone model to predict stable growth and residual strength for panels with MSD cracks. Their method was able to predict residual strength within 9% of their experimental results. Even though this method is analytical, it is not as easy to use for quick calculations as the linkup model and its modifications. As a matter of fact, the experimental data for 11 of the MSD crack configurations tested by Xu et al. [19] are used in this current investigation. Probably, it is worth mentioning that for these 11 crack configurations, the residual strength predictions of the modified linkup model (WSU2) are more accurate than those of the Xu et al. [19] method (the mean absolute errors for the WSU2 model and the Xu et al. method are 4% and 7%, respectively).

In addition to the typical fracture mechanics approaches mentioned earlier, some attempts were also made to use data-driven methods for predicting the residual strength of panels with MSD. Wu et al. [20] used a load-sharing parallel system reliability model for residual strength assessment. Monte Carlo simulation was used to derive the residual strength probability distribution, and they verified their approach by comparing to test results, and the relative error was about 10%. Finally, Pidaparti et al. [21,22] used ANN and inverse ANN mapping for predicting the residual strength of aluminum panels with MSD and corrosion thinning. Experimental data of about 40 panels, obtained from the literature, were used for training the network. However, it should be noted that the group of panels used for training included both unstiffened and stiffened panels, although none of the used ANN inputs accounted for the stiffened panels. It is not clear how stiffened panels data were handled, while none of the inputs accounted for this different configuration. It is likely that they were included to increase the number of data points used for ANN training. The ANN was tested using a selected group of nine unstiffened panels (no stiffened panels were used for testing), and the reported mean absolute error for the ANN residual strength prediction was 12%. As a matter of fact, such an error level is relatively high compared to that of some of the other engineering and computational methods reported in the literature [15].

### 2.2. Applications of ANN in Fracture Mechanics

The review conducted by Nasiri et al. [34] concluded that among the five artificial intelligence techniques of the Bayesian network, ANN, genetic algorithms, fuzzy logic, and case-based learning, ANN has attracted most of the researchers’ interest in fracture mechanics applications and resulted in the most sufficient accuracy. Nasiri et al. classified the ANN applications in fracture mechanics and mechanical fault detection into four sub-domains: failure mode and mechanism identification, damage and failure detection and diagnosis, fault and error detection and diagnosis, and mechanical fracture and fracture parameters. Balcıoğlu et al. [35] investigated the effects of bonding angle, patching type, and patching structure on the failure load of pultruded composite using different ANN algorithms. Bayesian regularization (BR), Levenberg–Marquardt (LM), and Scaled Conjugate Gradient (SCG) algorithms demonstrated high effectiveness in predicting the failure load of adhesively bonded composites. Hakim and Razak [36] proposed ANN to predict the damage severity of a steel girder bridge. The inputs of the network were the first five natural frequencies, while the output parameter was a damage index. In conclusion, this study demonstrated the ability of ANN to estimate the severity of damage with a maximum error of 6.8%. Janssens et al. [37] proposed a feature-learning approach for bearing fault detection based on convolutional ANN and compared it with the feature-engineering approach. The results of convolutional ANN outstandingly outperformed the traditional feature-engineering approach with respective accuracies of 93.6% and 87.3%. Shu et al. [38] developed a Back-Propagation Neural Network (BPNN) damage detection procedure, considering single and multi-damage cases for a one-span simply supported beam railway bridge. The ANN inputs considered in this study were the statistical properties of structural dynamic responses. The results revealed that ANN can accurately predict the damage severity and location. Feng et al. [39] used deep neural networks (DNN) to predict solidification cracking in metals using a relatively small dataset. In order to improve the performance of the DNN, as compared to the traditional shallow (single hidden layer) networks, they proposed the use of pre-trained and fine-tuned DNNs. Their results demonstrate that their approach shows a better generalized performance over shallow neural network and DNN trained by conventional methods.

Applications of ANN in fracture mechanics are mostly concerned with crack propagation, fatigue life, and failure mode prediction. Nechval et al. [40] adopted three-layer BBNN, with crack length as a single input and cyclic loading times as an output, to monitor fatigue crack growth. The feasibility and the ease of use of the model were verified on a special austenitic stainless steel material. Gajewski and Sadowski [41] adopted ANN, served by an FEA model for input data, to evaluate the behavior of a bituminous layered structure pavement material and estimate its crack propagation sensitivity. The study confirmed the effectiveness of the ANN–FEA model to monitor cracking in road pavements and indicated other application possibilities in similar layered materials. Lee et al. [42] showed that ANN can be accurately used to predict fatigue lives for the composite materials of five carbon and one glass fiber-reinforced plastics laminates. The most suitable inputs to the network were the minimum stress, peak stress, and the failure probability level. Hamdia et al. [43] predicted the fracture energy of polymer nanocomposites using ANN and adaptive neuro-fuzzy inference system (ANFIS) models. When comparing the performance evaluation indices for the proposed models with linear regression and literature models, the ANN and ANFIS models were found to be much more efficient. Mohanty et al. [44] designed ANN for fatigue life prediction considering different retardation parameters under constant amplitude for two aluminum alloys. In comparison with experimental data, the proposed ANN model was able to predict the fatigue life with a maximum error of 4%. Mortazavi and Ince [45] developed a radial basis function ANN model for predicting the fatigue crack growth behavior for short and long crack regimes. Experimental data for three different materials were used for training and validating the ANN model. Their results indicate that the ANN model has good interpolation capability. However, the model’s ability to extrapolate out of the training data range is poor, and the model effectiveness is greatly dependent on sufficient available input data. Additionally, ANN was also used in fracture parameters prediction. Seibi and Al-Alawi [46] employed ANN in predicating the facture toughness of beams and plates made of aluminum alloy under uniaxial and biaxial loading. The results demonstrated the capability of ANN to predict fracture toughness under various conditions with high accuracy. Moreover, the study revealed the relationship between the fracture toughness and the crack geometry, product dimensions, and working temperature. Ince [47] used an ANN model built directly from experimental data for predicting the fracture parameters of concrete. Two fracture parameters, stress intensity factor and crack tip opening displacement, were predicted by the ANN model, and the predictions were of reasonable accuracy.

ANN applications associated with fracture mechanics in aircraft structures are relatively scarce. Pidaparti and Palakal [48] presented an ANN method to represent the fatigue crack growth behavior under spectrum loading in aluminum panels. The inputs were characteristics related to the spectrum loading and crack growth, while the output was the corresponding loading cycles. Later, the same authors introduced an ANN-optimization system to estimate the fatigue life and fatigue crack growth for panels with MSD [49]. Two ANN models were combined: the BPNN model for predicting the local crack growth and an ANN optimization model to predict the overall behavior of the panel. For the optimization model, it was able to determine how fast cracks propagate and how long it takes for panels to experience fatigue for a given crack size. The comparison of the proposed system’s results with the actual fatigue test data illustrated the system’s ability to predict the crack growth and panel failure with fair accuracy. Spear et al. [50] described an ANN surrogate model combined with the FEM simulation framework for predicting the residual strength of flight structures. Four damage parameters, as inputs, and residual strength, as an output, were used to train and test the adopted feedforward BPNN. In their work, FEM crack growth simulations were used to derive the residual strength values, and these data was used for training the ANN, rather than using experimental data. The coupled proposed methodology provided useful means for achieving more adaptive aircraft control. Candelieri et al. [51] used ANN for the diagnosis and prognosis assessment of the structural health of aircraft. Mainly, the diagnosis is related to crack detection in terms of size and location identification—that is, if cracking has occurred in the bay or stringer components. For the prognosis, it aimed at estimating the evolution of the crack and the remaining useful life. The data used to build the ANN models were generated using FEM simulations. The proposed approach was found to be useful as an online monitoring and assessment system on aircraft.

## 3. Experimental Data

The experimental data used in this investigation were obtained from several sources in the literature [7,8,9,10,11,13,14,19]. In these experiments, the residual strength was obtained by performing a tensile test on relatively large-scale panels containing a lead crack and adjacent MSD cracks. The tests were conducted under displacement control mode to prevent the complete failure of the test panel. The reported residual strength values correspond to the loading level that caused the failure of the ligament between the lead crack and the adjacent MSD cracks on both sides. The stress value used in this investigation is the nominal remote stress, which is the load divided by the panel’s nominal cross-sectional area (that includes the stiffeners’ cross-sectional area in case of the stiffened panels). Reporting the stress rather than the load value is actually more meaningful here, since panels of different widths, thicknesses, and stiffener configurations are included in the experimental data.

As a matter of fact, testing panels with MSD cracks is not a quick and easy experimental task, and it is usually a time-and-cost-intensive process in both preparation and testing. For instance, the panels need to be relatively wide to avoid the dominance of the NSY type of failure and in order to resemble the actual case of aircraft structures realistically. For this reason, it is not possible to find large experimental datasets for panels with MSD in the literature. The most extensive testing program for the residual strength of panels with MSD was carried out by Smith et al. [9,10,11,12,13,14,15] over the course of several years. All this data are used in this study, and in addition to that, data from three other literature sources [7,8,19] that have a reasonable number of different cracks configurations (about ten or so) are also included to cover as many different configurations as possible.

The experimental data used in this investigation include a wide variety of panel configurations, materials, material conditions, widths, thicknesses, and lead and MSD cracks geometries. The experimental data presented here are grouped according to the panel configuration into three general groups: unstiffened panels, stiffened panels (it includes two different configurations), and lap-joint panels. The data for each of the three groups are presented in each of the succeeding subsections.

### 3.1. Unstiffened Panels

The unstiffened panel is the simplest panel configuration that can be tested for the effect of MSD cracks, and therefore, it is the most commonly found configuration in the literature. The test panels’ configuration is generally similar to that shown in Figure 1. Though not shown in the figure, anti-buckling fixtures are used in the experiments to prevent crack-face buckling. The unstiffened panels experimental data include three different aluminum sheet materials that are commonly used in aircraft (2024-T3, 2524-T3, 7075-T6), different panel widths (from 508 to 2286 mm), different sheet thickness (from 1 to 1.8 mm), bare and clad material conditions, longitudinal and transverse grain orientations, different lead crack lengths (from 76 to 546 mm), different MSD crack lengths (from 7.6 to 25.4 mm), and different ligament lengths between the lead and adjacent MSD cracks (from 3.8 to 38 mm). Some of the tested panels had cracks that were produced using an Electromagnetic Discharge Machine (EDM), while other panels had cracks that were produced by saw cut (fine jewelers saw); however, that information is not reported here, since previous studies have shown that the EDM and jewelers saw cracks give comparable results [9]. In addition, for some of the reported configurations, a number of duplicates were tested; however, the value being used here is the average value, since the variations in residual strength between the duplicates are not significant. The unstiffened panels data are presented in three tables according to the sheet material. Each table contains the material properties, panel and cracks geometry, and the experimentally obtained value of residual strength. It should be clearly stated here that the material properties reported in the table—namely yield strength and fracture toughness—are the standard handbook values (obtained according to material type, condition, grain orientation, and thickness), not the experimental value of the actual sheets used in the tests. Although many of the literature sources make tensile tests for the sheets used in the MSD experiments and report the yield strength, the handbook values are used here because this is the more realistic engineering approach. The yield strength values given in all the following tables are the A-basis values obtained from MIL-HDBK-5H [53]. Table 1 gives the experimental data for the Al 2024-T3 unstiffened panels, and it includes 50 unique configurations that are obtained from four different literature sources: WSU [9], NIST [8], FM [7] and SJTU [19]. It should be noted here that the SJTU experimental data (Xu et al. [19]) included in the table are only for the configurations with a lead crack and one MSD crack on either side (since the configurations with non-uniformly spaced MSD cracks do not represent realistic cases of MSD cracks as in aircraft structures). Configurations from U-1 to U-30 have MSD cracks emerging from the sides of holes (similar to Figure 1), while the remaining configurations have only MSD cracks (with very small pilot holes). Previous experience has shown that the effect of the hole size on residual strength is very minor, and therefore, the hole size is not included in the table nor in the ANN model. The loading direction relative to grain orientation is indicated in the table using (L-T) for loading in the longitudinal grain direction and (T-L) for loading in the transverse grain direction. Table 2 gives the experimental data for the Al 2524-T3 unstiffened panels, and it includes 22 unique configurations that are identical to the first 22 configurations in Table 1 (except that it is for a different material). It should be noted that the 2024-T3 and the 2524-T3 materials have the same yield strength but differ in fracture toughness, and the 2524-T3 (which is the newest material and it is currently used in aircraft industry) has more resistance to failure due to MSD cracks, as can be seen from the test data. Finally, Table 3 gives the experimental data for the Al 7075-T6 unstiffened panels, and it includes 12 unique configurations.

### 3.2. Stiffened Panels

**Figure 2:**
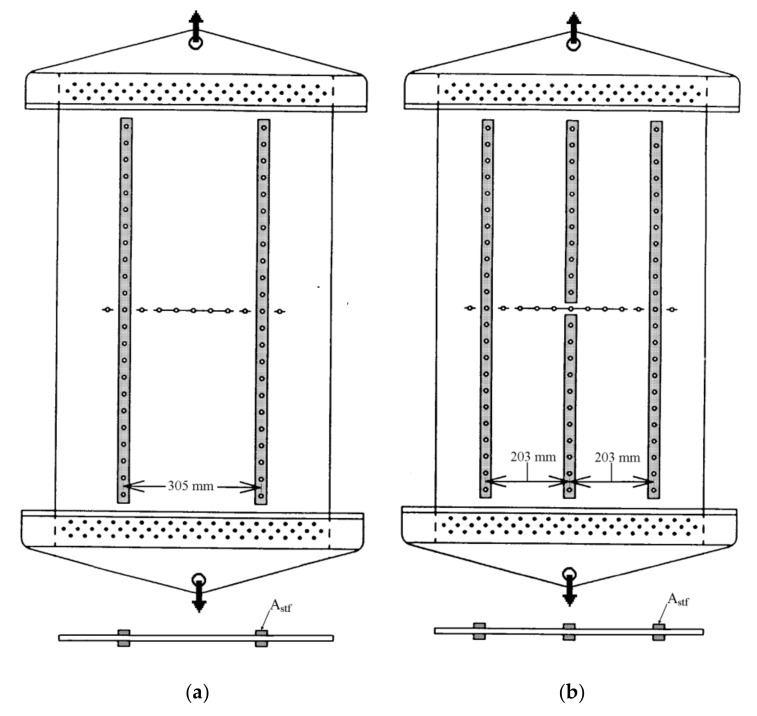
The stiffened panels’ configurations: (**a**) one-bay panel, (**b**) two-bay panel with broken middle stiffener.

The experimental data for the stiffened panels included two general configurations that are of interest in the aircraft industry, which are a lead crack centered between two stiffeners and a lead crack centered under a broken stiffener. Figure 2 shows the shape of the test panel used for each of the two different stiffened panel configurations. Anti-buckling fixtures were used in the experiments to prevent crack-face buckling, but they are not shown in the figure. Table 4 gives the experimental data for the stiffened panels where it includes 36 unique configurations all of the same material (Al 2024-T3 clad) and width but with two different stiffeners configurations [10]. In addition to the material and geometric parameters information of the unstiffened panels (in the previous subsection), this table also includes the cross-sectional area of each of the stiffeners (A_stf_) which are also made of aluminum and fixed at the front and back sides of the panel using bolts, as seen in the figure. For the first stiffened panel configuration (one-bay panels, from S-1 to S-21), three different sets of stiffeners were used in the experiments, where each set has a different cross-sectional area, as can be seen in the table.

### 3.3. Lap-Joint Panels

**Figure 3:**
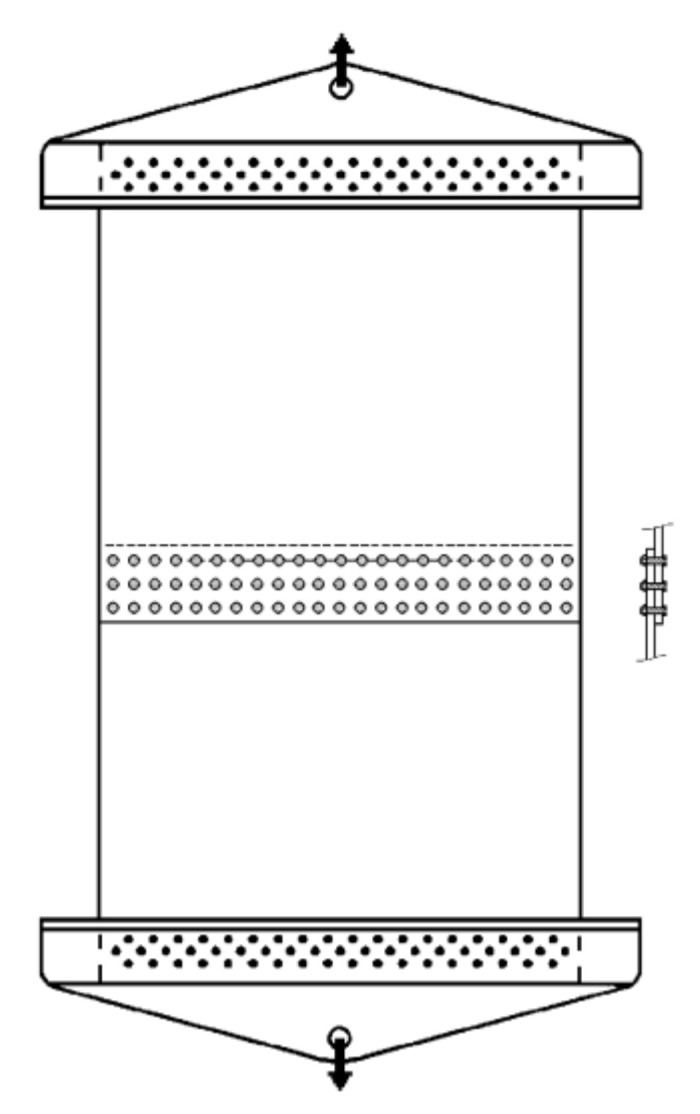
The bolted lap-joint panel configuration.

The experimental data for the lap-joint panels includes 27 unique configurations, all of the same material (Al 2024-T3 clad), grain orientation, width, and thickness [11]. Figure 3 shows the configuration of the test lap-joint panels where the two overlapping sheets are fixed together using three rows of bolts, as seen in the figure. Anti-buckling fixtures were used in the experiments to prevent crack-face buckling but are not shown in the figure. The experimental data for the 27 different crack configurations are given in Table 5.

## 4. ANN Modeling Procedure

The main objective of this study is to develop a single ANN model that can accurately predict the residual strength of panels with MSD for any panel configuration and geometry, material type and condition, and lead and MSD cracks geometry. Several parameters such as geometry, material properties, and panel configuration identifiers are used as inputs to the ANN. Only one hidden layer is used in the ANN structure, since the number of input/output nodes is relatively small. In addition, the use of ANN with one hidden layer (shallow network) generally gives better performance than multi-hidden-layer ANN (deep network) when dealing with small datasets, such as the case being considered here [39]. The learning algorithm, activation function, and the number of nodes in the hidden layer are optimized to give the best possible accuracy. It is important to mention again that each of the 147 data points used in here is unique, as no duplicates are being included. For each of the data groups corresponding to the different materials and panel configurations, the percentages of data points used for training, validation, and testing are about 66%, 17%, and 17%, respectively. Therefore, for each of the different groups of panels given by Table 1, Table 2, Table 3, Table 4 and Table 5, the number of data points used for training/validation/testing are 34/8/8 for the unstiffened 2024-T3 panels, 14/4/4 for the unstiffened 2524-T3 panels, 8/2/2 for the unstiffened 7075-T6 panels, 24/6/6 for the stiffened 2024-T3 panels, and 17/5/5 for the lap-joint 2024-T3 panels. This makes the total number of data points used for training (from all the different groups) to be 97 data points and the number of data points in the validation and testing datasets to be 25 data points each. 

To avoid any bias in the results that might come from choosing particular data points for testing the network, the individual data points that are used for training, validation, and testing are chosen randomly. Furthermore, to get more reliable results and robustly evaluate the ANN residual strength prediction accuracy, a total of 40 random combinations of data points are used for training, validation, and testing. This approach of choosing several random combinations is usually referred to as cross-validation, and it is commonly used in data-driven analyses [55]. In other words, the cross-validation procedure involves randomly sampling the training and validation observations with fixed fractions (66% and 17%, respectively, are being used here), where the remaining observations will be used as the testing dataset (17%). Then, the process is repeated a given number of times (40 times is being used here) using different observations for training and validation datasets. The entire ANN model development is repeated for each of the random training/validation/testing dataset combinations. This means that for our case, 40 different ANNs are developed using the 40 different random training datasets. Then, the performance of each of these 40 ANNs is evaluated using the testing dataset corresponding to the training dataset used for developing that particular ANN (i.e., the testing data points used with each of the 40 ANNs are never seen before by that particular ANN). It should be clearly stated here that the performance metrics reported in this paper are calculated by averaging 40 different values corresponding to the 40 different random combinations. So if we, for instance, consider the reported MAE_P_ value, it is calculated by averaging 40 MAE_P_ values of the 40 different random combinations used in the simulation trials.

### 4.1. Ann Inputs and Structure

**Figure 4:**
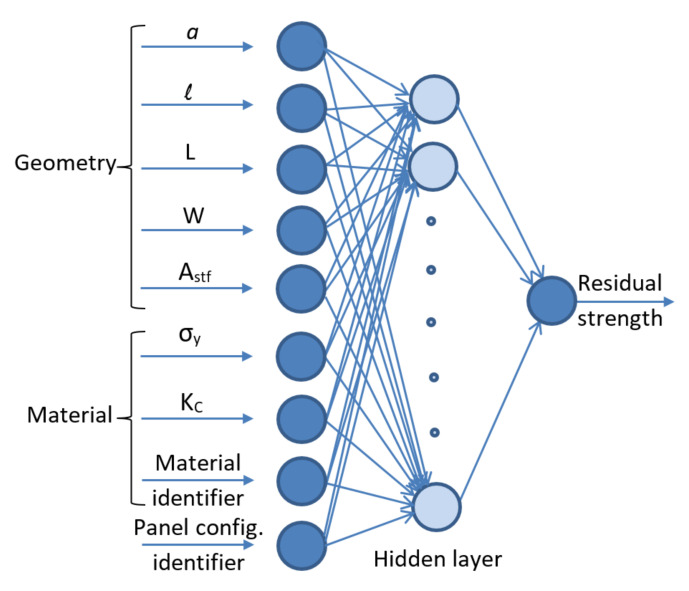
The artificial neural networks (ANN) structure.

A schematic illustration of the structure of the implemented ANN model is shown in Figure 4. The network consists of an input layer with nine input nodes where five inputs are geometry related, three inputs are material related, and one input is used to identify the configuration of the test panel. The geometric input parameters are the lead crack half-length, MSD cracks half-length, ligament length, panel width, and the stiffener cross-sectional area. As for the stiffener cross-sectional area (A_stf_), it will have a value for the stiffened panels only, while its magnitude will be zero for the unstiffened and lap-joint panels. As a matter of fact, the geometric input parameters being used here are carefully chosen based on the researchers’ experience with this type of fracture mechanics problems and in order to account for the experimental data, which are obtained from different sources. For the material input parameters, two material properties that have significance in fracture mechanics problems are used; these are the yield strength and fracture toughness. In addition to these two material properties, a material identification number is used to designate each of the three different materials being included here. Identification numbers 1, 2, and 3 are assigned to the 2024-T3, 2524-T3, and 7075-T6 aluminum alloys, respectively. A preliminary sensitivity analysis is performed to evaluate the significance of each of the ANN inputs and to see how it affects the residual strength. The sensitivity analysis is done by calculating the Pearson linear correlation coefficient between each of the inputs and the residual strength [56]. Although the sensitivity analysis has shown that the material identification number has a minor effect on residual strength as compared to the yield strength and fracture toughness, however, the material identification number is still included as one of the inputs mainly to designate the materials in case other materials are to be added later. For thin aluminum sheets, the yield strength and fracture toughness values depend on the material type, condition, and grain orientation, as well as sheet thickness. It should be noted here that sheet thickness is not included in the input parameters since both the yield strength and fracture toughness depend on the thickness; therefore, the thickness effect is already accounted for indirectly through these two material properties. Additionally, the fact that the residual strength being used here is the failure stress value rather than the failure load value makes the inclusion of the thickness among the ANN inputs unnecessary. The last input parameter is the panel configuration identification number, which is used to designate each of the different test panel configurations. The experimental data used in this investigation included four distinct test panel configurations. Therefore, identification numbers from 1 to 4 are assigned to distinguish the different configurations where 1: unstiffened panel, 2: one-bay stiffened panel, 3: two-bay stiffened panel with broken stiffener, and 4: lap-joint panel.

### 4.2. ANN Optimization

The validation datasets are used for optimizing the ANN’s configuration to give the best performance (i.e., accurate residual strength predictions), where the performance is evaluated using two metrics: MAE_P_ and RMSE_P_. Although the MAE_P_ is more meaningful for reflecting the level of the error, the two metrics are used to get a better understanding of the performance, since the RMSE_P_ reflects the closeness of the errors to the mean value. The MAE_P_ and RMSE_P_ are calculated here as:

(5) $${\mathrm{MAE}}_{P}=\frac{1}{N}\sum_{i=1}^{N}{\left|\frac{\sigma_{\mathrm{Prdct}}-\;\sigma_{\mathrm{Exp}}}{\sigma_{\mathrm{Exp}}}\;\%\right|}_{i}$$

(6) $${\mathrm{RMSE}}_{P}=\sqrt{\frac{1}{N}\sum_{i=1}^{N}{\left(\frac{\sigma_{\mathrm{Prdct}}-\;\sigma_{\mathrm{Exp}}}{\sigma_{\mathrm{Exp}}}\;\%\right)}_{i}{}^{2}}$$

The optimum ANN configuration is determined by inspecting the average MAE_P_ value for the 40 different random datasets combinations. The configuration of the ANN model is optimized in terms of the following:

(1) The adopted back-propagation learning algorithm ($l_{a}$) used to optimally define the ANN’s internal parameters (i.e., weights and biases). Basically, the weights and biases of the ANN are initially set randomly and then updated iteratively by calculating the error on the training outputs and distributing it back to the ANN layers.
(2) The hidden nodes activation function (f) used to process the ANN’s inputs.
(3) The number of nodes (H) in the hidden layer.

For this purpose, a robust, comprehensive search procedure is followed by considering:

- Three different possible learning algorithms; $l_{a}$= Bayesian Regularization (BR), Levenberg– Marquardt (LM), and Scaled Conjugate Gradient (SCG).
- Twelve different possible activation functions; f= ‘logsig’, ‘tansig’, ‘purelin’, ‘tribas’, ‘radbas’, ‘elliotsig’, ‘hardlims’, ‘hardlim’, ‘poslin’, ‘radbasn’, ‘satlin’, ‘satlins’.
- Up to 30 possible number of hidden nodes; H= [1–30].

For the activation functions, all the 12 different activation functions available in the MATLAB ANN environment are used in the optimization [57]. The difference between these functions lies in the way that each function calculates the layer’s output from the received inputs. As for the leaning algorithms, although more learning algorithms are available, the three algorithms used in the optimization (BR, LM, and SCG) are the most commonly used in the literature, and they are known to give good performance in comparison to the other available learning algorithms in different industrial applications [58,59,60,61]. The difference between the different learning algorithms lies in the way that each algorithm sets the internal parameters of the ANN (i.e., weights and biases). It is worth mentioning here that the modeling of the ANN has been conducted using a code that has been in-house developed in MATLAB environment.

## 5. Results and Discussion

In this study, ANN modeling is used for predicting the residual strength of aluminum panels with MSD cracks. A total of 147 experimental data points that represent 147 unique configurations of aluminum panels with MSD cracks are used here (97 for training, 25 for validation, and 25 for testing). These 147 data points represent different material types and conditions, panel configuration and geometry, cracks geometry, etc. Nine independent inputs are used for the ANN model where these inputs cover the different panel and cracks geometry, the different materials, and the different panel configurations. It is needless to say that the proper selection of the input parameters and how the different configurations are handled is essential to obtain good results. Overall, the ANN model has demonstrated good efficiency in predicting the residual strength for panels with MSD, as will be shown in the following sections.

### 5.1. Training Datasets Selection

As mentioned previously, a total of 97 data points (out of the 147 total) are used for ANN training, where these include 34 unstiffened 2024-T3 panels, 14 unstiffened 2524-T3 panels, 8 unstiffened 7075-T6 panels, 24 stiffened panels, and 17 lap-joint panels. These data points are chosen randomly, and 40 different random combinations of the 97 training data points are used in this investigation. This randomized selection procedure is meant to ensure the credibility of the obtained ANN results. However, by inspecting the individual data points within the 40 randomly selected combinations, it is noticed that occasionally, some of the data points that represent the upper or lower limit values of some of the important inputs (such as the crack length or ligament length) are not included in the training dataset. Therefore, in order to avoid any extrapolation in the ANN residual strength predictions (for the testing dataset), some fixed manually selected data points are always included in the training datasets. For each group of data points (Table 1, Table 2, Table 3, Table 4 and Table 5), the fixed manually selected data points included the upper and lower limits of each of the input parameters (e.g., ligament length, lead crack length, MSD crack length, etc.). The number of these fixed, manually selected, training data points for each of the different groups are 10 (out of 34) for the unstiffened 2024-T3 panels, 6 (out of 14) for the unstiffened 2524-T3 panels, 4 (out of 8) for the unstiffened 7075-T6 panels, 8 (out of 24) for the stiffened 2024-T3 panels, and 4 (out of 17) for the lap-joint 2024-T3 panels. Other than the fixed, manually selected, data points, the remaining data points for training as well as the validation and testing datasets are selected randomly. A comparison of the ANN results for this approach (with some fixed, manually selected, data points) to the completely randomized selection approach showed that it improves the accuracy of the residual strength predictions of the testing datasets. The results have shown that using these fixed data points among the training datasets (32 out of 97) improves the MAE_P_ by about 0.2%. Accordingly, this partially randomized training dataset’s selection approach is adapted in here. It should be mentioned here that the MAE_P_ improvement that results from using the partially randomized approach seems to be small (only 0.2%) because the MAE_P_ values are averaged for 40 different random combinations (as mentioned previously). However, for some particular data points, the error in the ANN residual strength predictions can be noticeable if the ANN is extrapolating out of the range used for training. It should also be indicated that this approach, which is being followed here to avoid the extrapolation for the ANN predictions, is consistent with the findings reported by Mortazavi and Ince [45] about the poor extrapolation ability of ANN.

### 5.2. Optimum ANN Configuration

**Figure 5:**
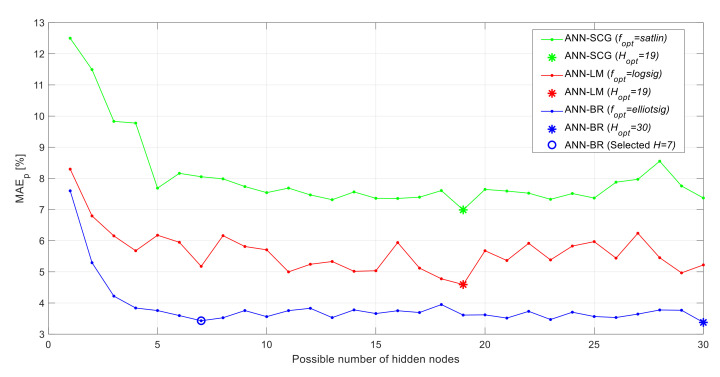
MAE_P_ evolution vs. the number of hidden neurons based on the validation dataset for the three learning algorithms (averages for the 40 random combinations of the validation dataset).

As mentioned previously, the ANN optimization is done using three different learning algorithms, 12 different activation functions, and up to 30 hidden nodes. The optimum ANN configuration is determined based on the MAE_P_ value (averaged over the 40 different random combinations). For identifying the best of the three learning algorithms (SCG, LM, BR), an ANN is developed using the training dataset based on each algorithm, and the validation dataset is used to determine the optimum configuration for each. Table 6 reports the best ANN configurations obtained for each learning algorithm in terms of the hidden nodes activation function ($f_{opt}$) and the number of hidden nodes ($H_{opt}$). The table shows that the BR learning algorithm outperforms the LM and SCG learning algorithms significantly in terms of all performance metrics: MAE_P_, RMSE_P_, and the coefficient of determination (R^2^). From the table, it can also be seen that the BR learning algorithm gives the best performance using the Elliot symmetric sigmoid (elliotsig) activation function along with 30 hidden nodes (based on the MAE_P_ value of the validation datasets). To further clarify the effect of the number of hidden nodes on the ANN performance, the evolution of the MAE_P_ versus the considered numbers of hidden nodes for each learning algorithm (using the optimum activation function) is shown in Figure 5. The figure shows that the BR learning algorithm continuously outperforms the two other learning algorithms for any number of hidden nodes. The optimum number of hidden nodes ($H_{opt}$), indicated by the asterisk in Figure 5, for each learning algorithm are those that minimize the MAE_P_ value using the validation datasets. The figure shows that for the BR algorithm, the optimum number of hidden nodes is 30, which corresponds to the lowest MAE_P_ value. By carefully inspecting the curve, it can be seen that the MAE_P_ value dropped rabidly at the beginning (at seven hidden nodes), and it remained relatively steady afterwards. Based on that, it can be seen that taking the number of hidden nodes to be seven would probably be good enough, and it will not make much difference in the residual strength prediction accuracy. As a matter of fact, some researchers use some rules of thumb for choosing the number of hidden nodes to be used in ANN models. One of the most commonly used rules of thump suggests that the number of hidden nodes should be somewhere in between the number of input nodes and the number of output nodes (i.e., between one and nine hidden nodes for our case) [62]. Based on this rule of thumb, taking the number of hidden nodes to be seven seems to be more reasonable, although our optimization results indicate that the 30 hidden nodes gives slightly lower MAE_P_ value (3.38% for 30 nodes vs. 3.43% for seven nodes). To further investigate that, the residual strength prediction performance of the seven hidden nodes and the 30 hidden nodes ANNs is also compared using the testing datasets (instead of the validation datasets that are used in the optimization). The compression based on the testing datasets (averages for the 40 random dataset combinations) shows that the seven hidden nodes gives slightly more accurate residual strength predictions where the MAE_P_ values using the seven and 30 hidden nodes are 3.82% and 3.92%, respectively. Consequently, the number of hidden nodes is taken to be seven, especially that such a small number of hidden nodes makes the ANN less complex and reduces the computational effort. Therefore, in summary, the ANN model that is being adopted in this study uses the “BR” learning algorithm, the “elliotsig” activation function, and seven nodes in the hidden layer. All the residual strength predictions presented in the succeeding sections are obtained using this ANN configuration.

### 5.3. ANN Residual Strength Predictions

In order to evaluate the performance of the ANN in predicting the residual strength of panels with MSD, the selected ANN configuration is applied on the testing “unseen” datasets, which include 40 different combinations, each consisting of 25 randomly selected data points. Of course, there are some differences in the calculated performance metrics for each of the 40 testing datasets. For instance, the testing dataset that gives the best performance has an MAE_P_ = 2.35%, and the testing dataset that gives the worst performance has an MAE_P_ = 6.35%, while the average MAE_P_ for the 40 testing datasets is 3.82%. This in fact shows the importance of using the cross-validation technique that makes the results more credible, since it eliminates the variations associated with the selection of the data points used in the testing dataset. It should be stressed here that the overall performance metrics reported in this paper are calculated by averaging over the 40 testing dataset combinations. 

**Figure 6:**
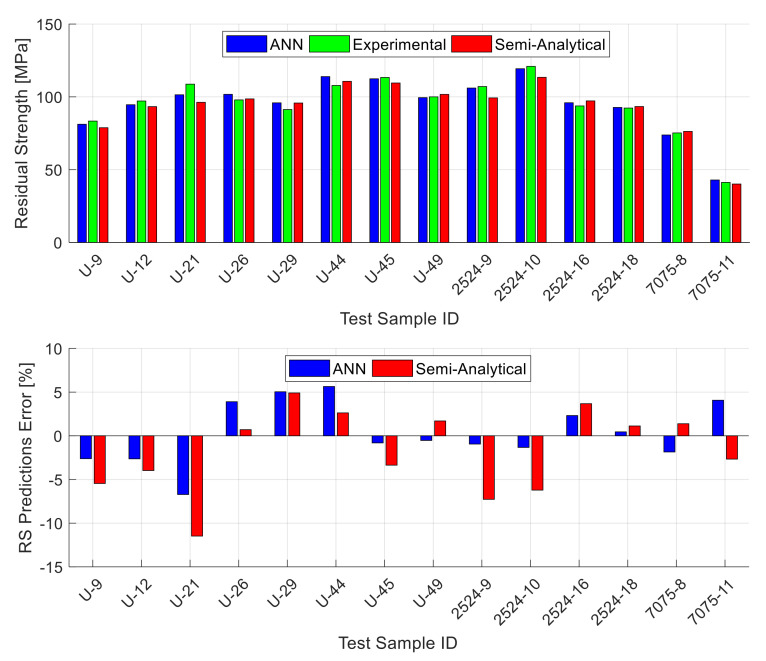
The unstiffened panels experimental residual strength values along with the predictions obtained by the ANN and the semi-analytical models (WSU2, MLU2524, and MLU7075) [9,13,14] for one of the testing datasets (**top**), together with the residual strength prediction errors (**bottom**). The shown ANN predictions are for the best of the 40 random combinations.

**Figure 7:**
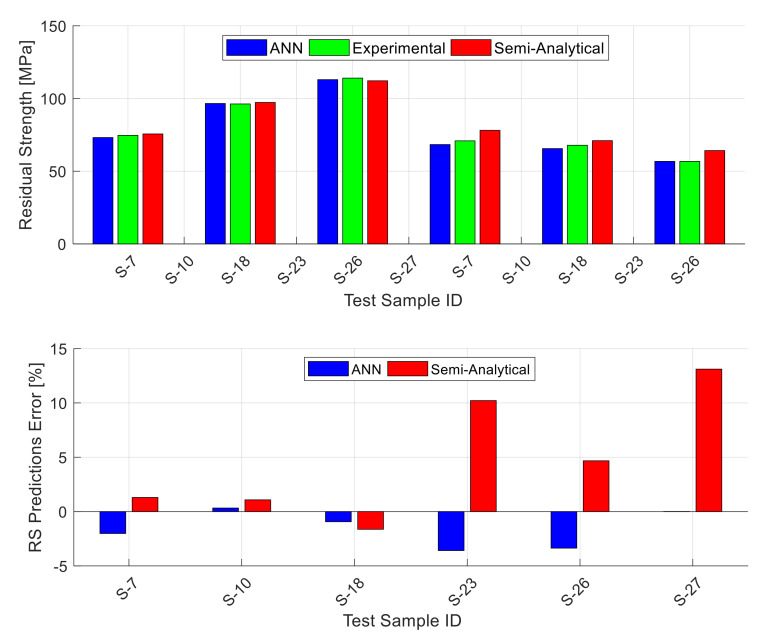
The stiffened panels experimental residual strength values along with the predictions obtained by the ANN and the semi-analytical model (WSU2) [10] for one of the testing datasets (**top**), together with the residual strength prediction errors (**bottom**). The shown ANN predictions are for the best of the 40 random combinations.

**Figure 8:**
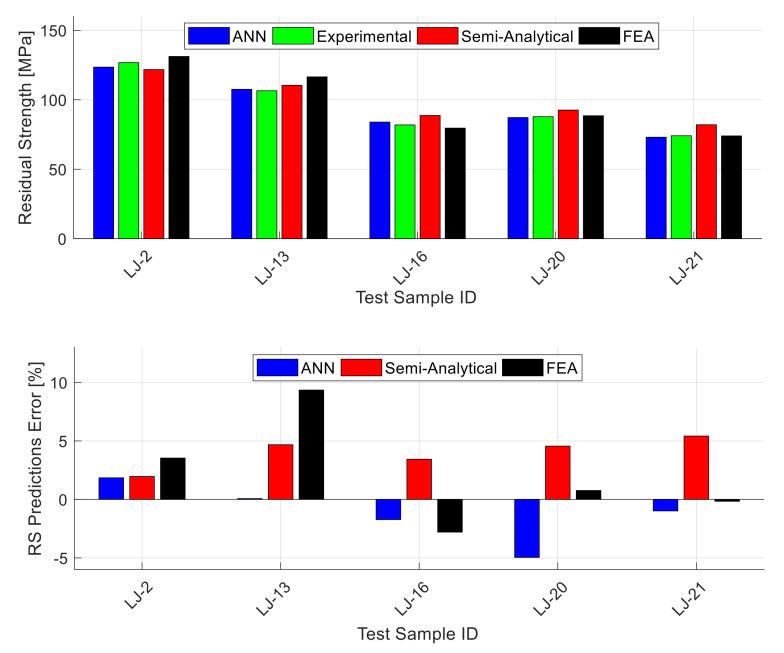
The lap-joint panels experimental residual strength values along with the predictions obtained by the ANN and the semi-analytical model (WSU2) [11] and FEM simulation [12] for one of the testing datasets (**top**), together with the residual strength prediction errors (**bottom**). The shown ANN predictions are for the best of the 40 random combinations.

Figure 6 shows exemplary results for one of the 40 testing datasets (the one that gives the best performance out of the 40 random testing datasets). The data shown in the figure is a subgroup of the 25 testing data points where this figure includes the unstiffened panels only (14 data points). The stiffened and lap-joint panels’ results are shown in separate figures. The upper part of Figure 6 shows the experimental residual strength value for each panel alongside with the ANN prediction and the prediction obtained using the semi-analytical model corresponding to the panel material (Equation (2) to Equation (4)). The lower part of the figure shows the magnitude of error in the predicted residual strength using the ANN and the semi-analytical model for each panel. The figure shows that the ANN residual strength predictions are fairly accurate and they generally outperform the semi-analytical model predictions in terms of accuracy. Figure 7 is similar to the previous figure, but it shows the stiffened panels’ subgroup (six data points) of the 25 testing data points. This figure shows that also for the stiffened panels, the ANN residual strength predictions are fairly accurate, and they generally outperform the semi-analytical model predictions in terms of accuracy. It is important here to mention that the stiffened panels included two different configurations (one-bay and two-bay with a broken stiffener) and the ANN residual strength predictions for both are of comparable accuracy. Finally, Figure 8 shows the results for the lap-joint panels’ subgroup (five data points) of the 25 testing data points. In this figure, in addition to the ANN and semi-analytical model predictions, the predictions obtained using elastic–plastic FEA based on CTOA criterion are also shown for comparison purposes. These FEA residual strength results for the lap-joint panels are obtained from Hijazi et al. [12]. This figure shows that also for the lap-joint panels, the ANN residual strength predictions are fairly accurate and they generally outperform both of the semi-analytical model and FEA predictions in terms of accuracy.

**Figure 9:**
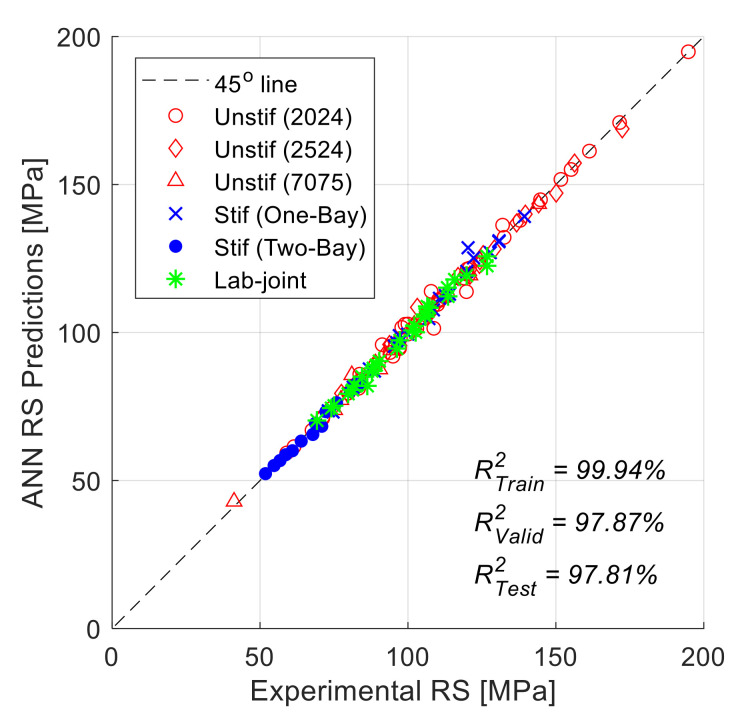
Correlation of ANN predictions with experimental residual strength values for all panels (the full 147 data points used for training, validation, and testing). The shown ANN predictions are for the best of the 40 random combinations.

In order to better visualize the overall accuracy for the ANN residual strength predictions, the correlation between the experimental results and ANN predictions for all the 147 data points is shown in Figure 9. As in the previous figures, the ANN results shown in Figure 9 are for the best one of the 40 random combinations of the testing datasets. The figure shows the results of all the 147 data points, which include the training (97), validation (25), and testing (25) data points. The figure shows that the ANN predictions are of very good accuracy, and there is no noticeable difference in accuracy for the different materials and panel configurations, since all the data are clustered close to the 45-degrees line. In addition, since the points are distributed uniformly above and below the 45-degrees line, it can be concluded that there is no over-or-under prediction tendency in the ANN predictions, and the prediction errors are fairly random (no systematic error is observed). The coefficient of determination (R^2^) values for the training, validation, and testing datasets are also shown in the figure. It should be noticed here that the coefficient of determination for the training dataset is clearly higher than those for the validation and training datasets. This is in fact quite expected, since the training data are already "seen" by the ANN (since they are used for training); therefore, the ANN can predict the residual strength for the training dataset more accurately that the "unseen" validation and testing datasets. The overall coefficient of determination for all the 147 data points shown in Figure 9 is 99.46%, which is another indicator of the goodness of the ANN predictions.

Finally, Table 7 gives the overall performance metrics calculated by averaging over the 40 random testing datasets. The table lists the MAE_P_ and RMSE_P_ values for the three different materials and for the three general panel configurations, as well as the totals for all materials and panel configurations. From the table, it is evident that the ANN predictions are generally accurate for all the deferent materials. However, it also can be seen that there are some relatively small differences in the residual strength predictions error levels between the different materials and panel configurations. The largest error is observed for the Al 7075-T6 material (MAE_P_ = 8.2%). The relatively high error associated with this material is rather expected, since the number of data points used for training the ANN is smaller than that for other materials (only eight data points are used for training). On the other hand, the best ANN performance is observed for the lap-joint panels where the MAE_P_ is 1.81%. This error value is lower than that observed for the unstiffened panels of the same Al 2024-T3 material (MAE_P_ = 4.61%), even though the number for panels used for ANN training for the unstiffened panels is larger than that for the lap-joint panels. In fact, this difference can be attributed to the fact that the unstiffened panels data have more variation where they include different thicknesses, material conditions, and grain orientations; while on the other hand, the lap-joint panels are all of the same thickness and grain orientation. Therefore, it is quite normal that the ANN is able to give better predictions for the lap-joint panels, since there is no variation in their material properties. It is probably worth mentioning here that using the actual values of the material properties (obtained by testing samples of the same sheet material) might slightly improve the ANN prediction accuracy. However, as mentioned previously, adopting the standard handbook values of the material properties is much more convenient for engineering use. By comparing the values of the two performance metrics given in the table, it can be seen that the RMSE_P_ values are consistently higher than the MAE_P_ values, but the difference is generally not very high. This difference between the RMSE_P_ and the MAE_P_ values comes from the variability of the error values for the individual predictions, and the fact that the difference is not very high indicates that the variability is not that significant. Finally, the table also shows the overall error for all the testing datasets where the MAE_P_ is equal to 3.82%. For the same group of testing datasets, the average error for the residual strength predictions obtained using the semi-analytical models (Equation (2) to Equation (4)) is 4.1%. Comparing the overall error values of the ANN and semi-analytical models predictions shows that both have the same level of accuracy. However, obtaining residual strength predictions using the ANN is easier, and the fact that a single ANN model is used for all materials and panel configurations makes it even more convenient.

## 6. Concluding Remarks

The presence of MSD cracks and their effect on residual strength is a serious concern for aging aircraft fleets. Several methodologies ranging from simple engineering models to sophisticated computational approaches can be used for estimating the residual strength of panels with MSD cracks. Data-driven techniques, and in particular ANN, have been successfully applied and are increasingly being used in a wide variety of engineering applications. In this paper, ANN modeling is used for predicting the residual strength of aluminum panels with MSD. The experimental data used for developing the ANN model (training/validation/testing) include 147 unique data points that cover several material types and conditions, panels and cracks geometry, and test panel configurations. The ANN model has nine independent inputs that deal with the geometry, materials, and panel configurations. The results presented in this paper demonstrate that the ANN model can predict the residual strength for all materials and configurations with high accuracy. The overall ANN model average prediction error (MAE_P_) is 3.82%, which puts it at the same accuracy level (and even better for many configurations) of the best available semi-analytical and computational approaches. The main conclusions of this study can be summarized in the following points:

- Proper selection of the input parameters for representing the different materials and the geometric and panel configurations is essential for obtaining good results using ANN. For instance, two fracture-related material properties (yield strength and fracture toughness) and a designation number are used as inputs to account for the three different materials being used here. The material properties also "indirectly" account for sheet thickness, material condition, and grain orientation, and this eliminates the need for some inputs. In addition, using the standard handbook values of the material properties (instead of the actual properties obtained from testing) makes the ANN modeling approach being used here simpler and more convenient for engineering use.
- Optimizing the ANN model is essential for obtaining high-accuracy predictions. The ANN optimization carried out here showed that there could be quite a significant difference in the prediction accuracy when using different learning algorithms, hidden node activation functions, and numbers of hidden nodes. In this investigation, the best ANN performance was obtained using the Bayesian Regularization learning algorithm with the Elliot symmetric sigmoid activation function and seven hidden nodes.
- In order to avoid bias and get more reliable results, it is essential to implement a randomized selection procedure for the data points used in the training, validation, and testing datasets; also, the randomized selection needs to be repeated several times (cross-validation). Additionally, it is beneficial to include some fixed, manually selected data points that cover the upper and lower limit values of the different inputs within the training group. This will avoid the extrapolation in the ANN predictions and thus improve the accuracy.
- There are some differences (relatively small yet noticeable) in the average residual strength prediction error values between the different materials and panel configurations. These differences in the error values can be attributed to two factors: (i) the different number of data points available for training the ANN for the different materials and configurations and (ii) the amount of variation in the different inputs parameters within the different materials and configurations. For instance, the highest error is observed for the 7075-T6 material (MAE_P_ = 8.2%) since only eight data points are used for training. In fact, eight data points is a very small number, and it is definitely not sufficient for training an ANN; however, the error level is still reasonable, since this material is not used alone for developing an ANN model but rather among a larger group of data points that share many common geometric and configuration inputs, although the materials are different. On the other hand, the best accuracy (MAE_P_ = 1.81%) is observed for the lap-joint panels, since they all share the same sheet thickness, material condition, and grain orientation.

## Figures and Tables

**Table 1:** Experimental data for the unstiffened 2024-T3 panel configurations [7,8,9,19].

Panel ID	Source	Mat. Cond.	Grain Orient.	σ_y_ MPa	K_C_ MPa.m^1/2^	t mm	W mm	*a* mm	*ℓ* mm	L mm	σ_Exp_ MPa
**U-1**	WSU	Clad	L-T	310.3	114.3	1.6	610	93.35	4.45	3.81	79.84
**U-2**	WSU	Clad	L-T	310.3	114.3	1.6	610	90.81	4.45	6.35	97.15
**U-3**	WSU	Clad	L-T	310.3	114.3	1.6	610	88.27	4.45	8.89	112.18
**U-4**	WSU	Clad	T-L	275.8	109.9	1.6	610	84.46	8.26	8.89	94.25
**U-5**	WSU	Clad	T-L	275.8	109.9	1.6	610	83.19	6.99	11.43	110.04
**U-6**	WSU	Clad	T-L	275.8	109.9	1.6	610	81.92	5.72	13.97	120.04
**U-7**	WSU	Clad	T-L	275.8	109.9	1.6	610	80.65	4.45	16.51	132.52
**U-8**	WSU	Clad	T-L	275.8	109.9	1.6	610	118.75	4.45	3.81	67.57
**U-9**	WSU	Clad	T-L	275.8	109.9	1.6	610	116.21	4.45	6.35	83.36
**U-10**	WSU	Clad	T-L	275.8	109.9	1.6	610	113.67	4.45	8.89	94.94
**U-11**	WSU	Clad	T-L	275.8	109.9	1.6	610	109.86	8.26	8.89	82.33
**U-12**	WSU	Clad	T-L	275.8	109.9	1.6	610	108.59	6.99	11.43	97.15
**U-13**	WSU	Clad	T-L	275.8	109.9	1.6	610	107.32	5.72	13.97	105.7
**U-14**	WSU	Clad	T-L	275.8	109.9	1.6	610	106.05	4.45	16.51	119.77
**U-15**	WSU	Clad	L-T	310.3	114.3	1.6	610	144.15	4.45	3.81	59.02
**U-16**	WSU	Clad	L-T	310.3	114.3	1.6	610	141.61	4.45	6.35	73.98
**U-17**	WSU	Clad	L-T	310.3	114.3	1.6	610	139.07	4.45	8.89	83.71
**U-18**	WSU	Clad	T-L	275.8	109.9	1.6	610	135.26	8.26	8.89	71.23
**U-19**	WSU	Clad	T-L	275.8	109.9	1.6	610	133.99	6.99	11.43	83.50
**U-20**	WSU	Clad	T-L	275.8	109.9	1.6	610	132.72	5.72	13.97	93.91
**U-21**	WSU	Clad	T-L	275.8	109.9	1.6	610	131.45	4.45	16.51	108.73
**U-22**	WSU	Clad	T-L	275.8	109.9	1.6	610	160.66	8.26	8.89	71.23
**U-23**	NIST	Bare	L-T	324.1	111.0	1.02	2286	254	6.35	6.35	61.50
**U-24**	NIST	Bare	L-T	324.1	111.0	1.02	2286	177.8	5.08	7.62	84.12
**U-25**	NIST	Bare	L-T	324.1	111.0	1.02	2286	71.12	7.62	10.16	137.9
**U-26**	NIST	Bare	L-T	324.1	111.0	1.02	2286	195.58	5.08	15.24	97.91
**U-27**	NIST	Bare	L-T	324.1	111.0	1.02	2286	241.3	6.35	19.05	88.95
**U-28**	NIST	Bare	L-T	324.1	111.0	1.02	2286	96.52	7.62	22.86	161.34
**U-29**	NIST	Bare	L-T	324.1	111.0	1.02	2286	273.05	6.35	25.4	91.29
**U-30**	NIST	Bare	L-T	324.1	111.0	1.02	2286	127	5.08	33.02	151.69
**U-31**	FM *	Clad	T-L	268.9	113.2	1.02	508	101.6	3.81	8.89	97.43
**U-32**	FM *	Clad	T-L	268.9	113.2	1.02	508	96.52	6.35	11.43	99.98
**U-33**	FM *	Clad	T-L	268.9	113.2	1.02	508	40.64	10.16	12.7	144.8
**U-34**	FM *	Clad	T-L	268.9	113.2	1.02	508	63.5	12.7	12.7	106.05
**U-35**	FM *	Clad	T-L	268.9	113.2	1.02	508	93.98	6.35	13.97	110.32
**U-36**	FM *	Clad	T-L	268.9	113.2	1.02	508	40.64	6.35	16.51	171.55
**U-37**	FM *	Clad	T-L	268.9	113.2	1.02	508	91.44	6.35	16.51	118.94
**U-38**	FM *	Clad	T-L	268.9	113.2	1.02	508	76.2	6.35	31.75	155.14
**U-39**	FM *	Clad	T-L	268.9	113.2	1.02	508	38.1	12.7	38.1	194.78
**U-40**	SJTU *	Clad	L-T	303.4	117.6	1	600	90	7.5	8	106.83
**U-41**	SJTU *	Clad	L-T	303.4	117.6	1	600	90	7.5	12	120.83
**U-42**	SJTU *	Clad	L-T	303.4	117.6	1	600	90	7.5	18	132
**U-43**	SJTU *	Clad	L-T	303.4	117.6	1	600	111	7.5	10	103
**U-44**	SJTU *	Clad	L-T	303.4	117.6	1	600	111	7.5	15	107.83
**U-45**	SJTU *	Clad	L-T	303.4	117.6	1	600	113	7.5	15	113.33
**U-46**	SJTU *	Clad	L-T	303.4	117.6	1	600	113	7.5	20	119.67
**U-47**	SJTU *	Clad	L-T	303.4	117.6	1	600	136	7.5	20	99
**U-48**	SJTU *	Clad	L-T	303.4	117.6	1	600	138	7.5	30	110.67
**U-49**	SJTU *	Clad	L-T	303.4	117.6	1	600	143	7.5	20	100
**U-50**	SJTU *	Clad	L-T	303.4	117.6	1	600	148	7.5	30	106.67

* Panels have MSD cracks with very small pilot holes.

**Table 2:** Experimental data for the unstiffened (Clad) 2524-T3 panel configurations [14].

Panel ID	Grain Orient.	σ_y_ MPa	K_C_ MPa.m^1/2^	t mm	W mm	*a* mm	*ℓ* mm	L mm	σ_Exp_ MPa
2524-1	L-T	310.3	204.4	1.6	610	93.35	4.45	3.81	102.11
2524-2	L-T	310.3	204.4	1.6	610	90.81	4.45	6.35	124.18
2524-3	L-T	310.3	204.4	1.6	610	88.27	4.45	8.89	139.69
2524-4	T-L	275.8	180.2	1.6	610	84.46	8.26	8.89	125.42
2524-5	T-L	275.8	180.2	1.6	610	83.19	6.99	11.43	144.17
2524-6	T-L	275.8	180.2	1.6	610	81.92	5.72	13.97	156.31
2524-7	T-L	275.8	180.2	1.6	610	80.65	4.45	16.51	172.44
2524-8	T-L	275.8	180.2	1.6	610	118.75	4.45	3.81	89.01
2524-9	T-L	275.8	180.2	1.6	610	116.21	4.45	6.35	107.08
2524-10	T-L	275.8	180.2	1.6	610	113.67	4.45	8.89	120.94
2524-11	T-L	275.8	180.2	1.6	610	109.86	8.26	8.89	108.67
2524-12	T-L	275.8	180.2	1.6	610	108.59	6.99	11.43	124.32
2524-13	T-L	275.8	180.2	1.6	610	107.32	5.72	13.97	136.73
2524-14	T-L	275.8	180.2	1.6	610	106.05	4.45	16.51	150.1
2524-15	L-T	310.3	204.4	1.6	610	144.15	4.45	3.81	77.5
2524-16	L-T	310.3	204.4	1.6	610	141.61	4.45	6.35	93.77
2524-17	L-T	310.3	204.4	1.6	610	139.07	4.45	8.89	103.15
2524-18	T-L	275.8	180.2	1.6	610	135.26	8.26	8.89	92.32
2524-19	T-L	275.8	180.2	1.6	610	133.99	6.99	11.43	107.63
2524-20	T-L	275.8	180.2	1.6	610	132.72	5.72	13.97	116.94
2524-21	T-L	275.8	180.2	1.6	610	131.45	4.45	16.51	129.21
2524-22	T-L	275.8	180.2	1.6	610	160.66	8.26	8.89	80.67

**Table 3:** Experimental data for the unstiffened (Bare) 7075-T6 panel configurations [13].

Panel ID	Grain Orient.	σ_y_ MPa	K_C_ MPa.m^1/2^	t mm	W mm	*a* mm	*ℓ* mm	L mm	σ_Exp_ MPa
7075-1	T-L	468.9	76.9	1.8	610	84.46	6.99	10.16	100.6
7075-2	T-L	468.9	76.9	1.8	610	109.86	8.26	8.89	81.02
7075-3	T-L	468.9	76.9	1.8	610	108.59	6.99	11.43	94.25
7075-4	T-L	468.9	76.9	1.8	610	108.59	5.72	12.7	96.94
7075-5	T-L	468.9	76.9	1.8	610	107.32	4.45	15.24	105.36
7075-6	T-L	468.9	76.9	1.8	610	133.99	5.72	12.7	81.22
7075-7	T-L	468.9	76.9	1.8	610	132.72	5.72	13.97	90.46
7075-8	T-L	468.9	76.9	1.8	610	135.26	6.99	10.16	75.22
7075-9	T-L	468.9	76.9	1.8	610	158.12	5.72	13.97	77.29
7075-10	T-L	468.9	76.9	1.8	610	191.14	8.26	3.81	32.61
7075-11	T-L	468.9	76.9	1.8	610	189.87	8.26	5.08	41.23
7075-12	T-L	468.9	76.9	1.8	610	188.60	8.26	6.35	46.68

**Table 4:** Experimental data for the stiffened (Clad) 2024-T3 panel configurations [10].

Panel ID	Stiff. Config.	Grain Orient.	σ_y_ MPa	K_C_ MPa.m^1/2^	A_stf_ mm^2^	t mm	W mm	*a* mm	*ℓ* mm	L mm	σ_Exp_ MPa
S-1	One-Bay	T-L	275.8	109.9	105	1.6	610	118.75	4.45	3.81	73.09
S-2	One-Bay	T-L	275.8	109.9	105	1.6	610	116.21	4.45	6.35	86.95
S-3	One-Bay	T-L	275.8	109.9	105	1.6	610	113.67	4.45	8.89	99.91
S-4	One-Bay	T-L	275.8	109.9	105	1.6	610	108.59	6.99	11.43	95.43
S-5	One-Bay	T-L	275.8	109.9	105	1.6	610	107.32	5.72	13.97	110.66
S-6	One-Bay	T-L	275.8	109.9	105	1.6	610	106.05	4.45	16.51	122.18
S-7	One-Bay	T-L	275.8	109.9	105	1.6	610	144.15	4.45	3.81	74.67
S-8	One-Bay	T-L	275.8	109.9	105	1.6	610	141.61	4.45	6.35	88.67
S-9	One-Bay	T-L	275.8	109.9	105	1.6	610	139.07	4.45	8.89	97.08
S-10	One-Bay	T-L	275.8	109.9	105	1.6	610	133.99	6.99	11.43	96.25
S-11	One-Bay	T-L	275.8	109.9	105	1.6	610	132.72	5.72	13.97	112.66
S-12	One-Bay	T-L	275.8	109.9	105	1.6	610	131.45	4.45	16.51	127.83
S-13	One-Bay	L-T	310.3	114.3	161.3	1.6	610	113.67	4.45	8.89	107.08
S-14	One-Bay	L-T	310.3	114.3	151.6	1.6	610	108.59	6.99	11.43	108.6
S-15	One-Bay	L-T	310.3	114.3	151.6	1.6	610	107.32	5.72	13.97	119.9
S-16	One-Bay	L-T	310.3	114.3	151.6	1.6	610	106.05	4.45	16.51	130.8
S-17	One-Bay	L-T	310.3	114.3	161.3	1.6	610	144.15	4.45	3.81	81.5
S-18	One-Bay	L-T	310.3	114.3	151.6	1.6	610	133.99	6.99	11.43	114.04
S-19	One-Bay	L-T	310.3	114.3	161.3	1.6	610	132.72	5.72	13.97	120.32
S-20	One-Bay	L-T	310.3	114.3	161.3	1.6	610	131.45	4.45	16.51	139.35
S-21	One-Bay	L-T	303.4	117.6	105	1.02	610	81.92	5.72	13.97	130.73
S-22	Two-Bay *	T-L	275.8	109.9	105	1.6	610	107.32	5.72	13.97	80.53
S-23	Two-Bay *	T-L	275.8	109.9	105	1.6	610	108.59	6.99	11.43	70.88
S-24	Two-Bay *	T-L	275.8	109.9	105	1.6	610	109.86	8.26	8.89	58.68
S-25	Two-Bay *	T-L	275.8	109.9	105	1.6	610	132.72	5.72	13.97	75.85
S-26	Two-Bay *	T-L	275.8	109.9	105	1.6	610	133.99	6.99	11.43	67.85
S-27	Two-Bay *	T-L	275.8	109.9	105	1.6	610	135.26	8.26	8.89	56.75
S-28	Two-Bay *	T-L	275.8	109.9	105	1.6	610	158.12	5.72	13.97	72.26
S-29	Two-Bay *	T-L	275.8	109.9	105	1.6	610	159.39	6.99	11.43	63.92
S-30	Two-Bay *	T-L	275.8	109.9	105	1.6	610	160.66	8.26	8.89	54.75
S-31	Two-Bay *	T-L	275.8	109.9	105	1.6	610	183.52	5.72	13.97	68.74
S-32	Two-Bay *	T-L	275.8	109.9	105	1.6	610	184.79	6.99	11.43	60.95
S-33	Two-Bay *	T-L	275.8	109.9	105	1.6	610	186.06	8.26	8.89	51.85
S-34	Two-Bay *	L-T	310.3	114.3	105	1.6	610	107.32	5.72	13.97	97.01
S-35	Two-Bay *	L-T	310.3	114.3	105	1.6	610	132.72	5.72	13.97	84.26
S-36	Two-Bay *	L-T	310.3	114.3	105	1.6	610	158.12	5.72	13.97	82.33

* Panels with crack centered under broken middle stiffener.

**Table 5:** Experimental data for the lap-joint (Clad) 2024-T3 panel configurations [11].

Panel ID	Grain Orient.	σ_y_ MPa	K_C_ MPa.m^1/2^	t mm	W mm	*a* mm	*ℓ* mm	L mm	σ_Exp_ MPa
LJ-1	T-L	268.9	109.9	1.42	610	106.52	3.65	16.83	126.73
LJ-2	T-L	268.9	109.9	1.42	610	106.52	4.92	15.56	115.77
LJ-3	T-L	268.9	109.9	1.42	610	107.79	3.65	15.56	126.73
LJ-4	T-L	268.9	109.9	1.42	610	107.79	4.92	14.29	113.35
LJ-5	T-L	268.9	109.9	1.42	610	107.79	6.19	13.02	106.53
LJ-6	T-L	268.9	109.9	1.42	610	109.06	3.65	14.29	119.84
LJ-7	T-L	268.9	109.9	1.42	610	109.06	4.92	13.02	113.56
LJ-8	T-L	268.9	109.9	1.42	610	109.06	6.19	11.75	105.77
LJ-9	T-L	268.9	109.9	1.42	610	109.06	7.46	10.48	101.49
LJ-10	T-L	268.9	109.9	1.42	610	131.92	3.65	16.83	107.29
LJ-11	T-L	268.9	109.9	1.42	610	131.92	4.92	15.56	102.6
LJ-12	T-L	268.9	109.9	1.42	610	133.19	3.65	15.56	105.91
LJ-13	T-L	268.9	109.9	1.42	610	133.19	4.92	14.29	97.29
LJ-14	T-L	268.9	109.9	1.42	610	133.19	6.19	13.02	90.32
LJ-15	T-L	268.9	109.9	1.42	610	134.46	3.65	14.29	102.18
LJ-16	T-L	268.9	109.9	1.42	610	134.46	4.92	13.02	96.25
LJ-17	T-L	268.9	109.9	1.42	610	134.46	6.19	11.75	88.26
LJ-18	T-L	268.9	109.9	1.42	610	134.46	7.46	10.48	81.91
LJ-19	T-L	268.9	109.9	1.42	610	157.32	3.65	16.83	89.29
LJ-20	T-L	268.9	109.9	1.42	610	157.32	4.92	15.56	86.26
LJ-21	T-L	268.9	109.9	1.42	610	158.59	3.65	15.56	87.84
LJ-22	T-L	268.9	109.9	1.42	610	158.59	4.92	14.29	81.29
LJ-23	T-L	268.9	109.9	1.42	610	158.59	6.19	13.02	75.02
LJ-24	T-L	268.9	109.9	1.42	610	159.86	3.65	14.29	84.46
LJ-25	T-L	268.9	109.9	1.42	610	159.86	4.92	13.02	79.78
LJ-26	T-L	268.9	109.9	1.42	610	159.86	6.19	11.75	74.12
LJ-27	T-L	268.9	109.9	1.42	610	159.86	7.46	10.48	69.23

**Table 6:** Optimum artificial neural networks (ANN) configuration (based the validation dataset) for each learning algorithm and its performance metrics (averages for the 40 random combinations of the validation dataset).

	SCG	LM	BR
**$f_{opt}$**	satlin	logsig	elliotsig
**$H_{opt}$**	19	19	30
**MAE_P_ [%]**	6.99	4.59	3.38
**RSME_P_ [%]**	9.61	7.22	4.9
**R^2^ [%]**	85.77	91.27	96

**Table 7:** The ANN predictions overall performance metrics for the different materials and panel configurations (averages for the 40 random combinations of the testing datasets).

	Unstif (2024)	Unstif (2524)	Unstif (7075)	Stif	Lab-joint	All
**MAE_P_** [%]	4.61	2.86	8.2	3.62	1.81	**3.82**
**RMSE_P_** [%]	6.36	3.38	9.96	4.49	2.21	**5.88**
